# Gouty Arthropathy: Review of Clinical Manifestations and Treatment, with Emphasis on Imaging

**DOI:** 10.3390/jcm11010166

**Published:** 2021-12-29

**Authors:** Jennifer S. Weaver, Ernest R. Vina, Peter L. Munk, Andrea S. Klauser, Jamie M. Elifritz, Mihra S. Taljanovic

**Affiliations:** 1 Department of Radiology, University of New Mexico, Albuquerque, NM 87131, USA; mihrat@radiology.arizona.edu; 2 Department of Medicine, University of Arizona Arthritis Center, Tucson, AZ 85724, USA; evina@arizona.edu; 3 Department of Radiology, University of British Columbia, Vancouver, BC V6T 1Z4, Canada; peter.munk@vch.ca; 4 Department of Radiology, Vancouver General Hospital, Vancouver, BC V5Z 1M9, Canada; 5 Radiology Department, Medical University Innsbruck, Anichstrasse 35, 6020 Innsbruck, Austria; andrea.klauser@i-med.ac.at; 6 Departments of Radiology and Pathology, University of New Mexico, Albuquerque, NM 87131, USA; jelifritz@salud.unm.edu; 7 New Mexico Office of the Medical Investigator, Albuquerque, NM 87131, USA; 8 Departments of Medical Imaging and Orthopaedic Surgery, University of Arizona, Tucson, AZ 85721, USA

**Keywords:** gout, monosodium urate crystals, crystalline arthropathy, imaging, magnetic resonance imaging, sonography, radiography, CT, dual energy CT, treatment

## Abstract

Gout, a crystalline arthropathy caused by the deposition of monosodium urate crystals in the articular and periarticular soft tissues, is a frequent cause of painful arthropathy. Imaging has an important role in the initial evaluation as well as the treatment and follow up of gouty arthropathy. The imaging findings of gouty arthropathy on radiography, ultrasonography, computed tomography, dual energy computed tomography, and magnetic resonance imaging are described to include findings of the early, acute and chronic phases of gout. These findings include early monosodium urate deposits, osseous erosions, and tophi, which may involve periarticular tissues, tendons, and bursae. Treatment of gout includes non-steroidal anti-inflammatories, colchicine, glucocorticoids, interleukin-1 inhibitors, xanthine oxidase inhibitors, uricosuric drugs, and recombinant uricase. Imaging is critical in monitoring response to therapy; clinical management can be modulated based on imaging findings. This review article describes the current standard of care in imaging and treatment of gouty arthropathy.

## 1. Introduction

**Figure 1:**
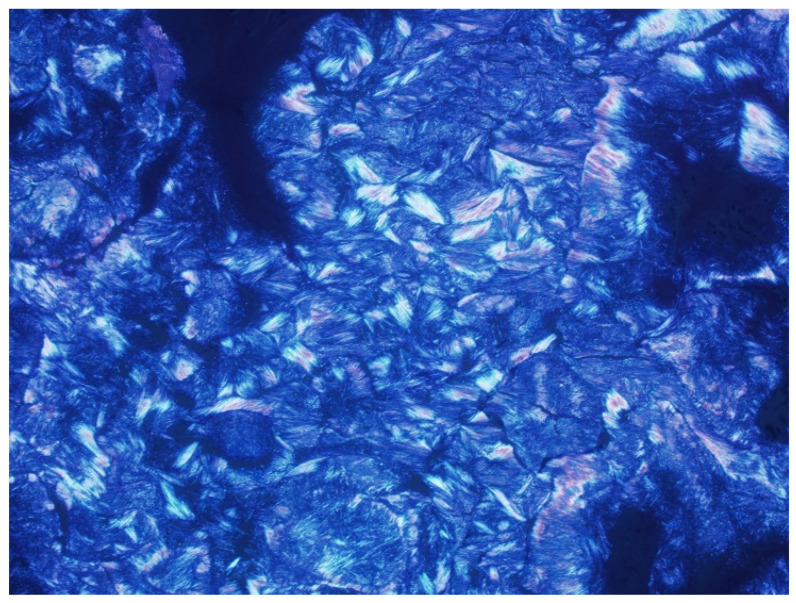
Image from polarizing microscopy 100× shows phagocytosed needle-shaped monosodium urate (MSU) crystals with strong negative birefringence. Image courtesy of Nadja Falk MD; Albuquerque, NM, USA.

Gout is the most common cause of inflammatory arthritis in adults [1,2,3,4,5,6,7,8,9,10,11,12,13]. It affects approximately 1–2% of the population of industrialized countries and is more common in older males [1,6,13,14,15,16,17,18,19,20,21]. Gouty arthropathy occurs secondary to abnormal purine metabolism, the end product of which is uric acid, combined with underexcretion or overproduction of uric acid, resulting in sustained hyperuricemia. Hyperuricemia, above the local solubility, can lead to monosodium urate (MSU) crystal deposition (Figure 1) in joints, on the surface of the hyaline cartilage and within periarticular soft tissues such as tendons, ligaments, retinacula, and bursae, with resulting inflammatory response [7,13,14,16,17,19,22,23,24,25,26,27,28,29,30,31,32]. Gout predominantly affects the peripheral joints, but the axial skeleton may also be affected [33].

**Figure 2:**
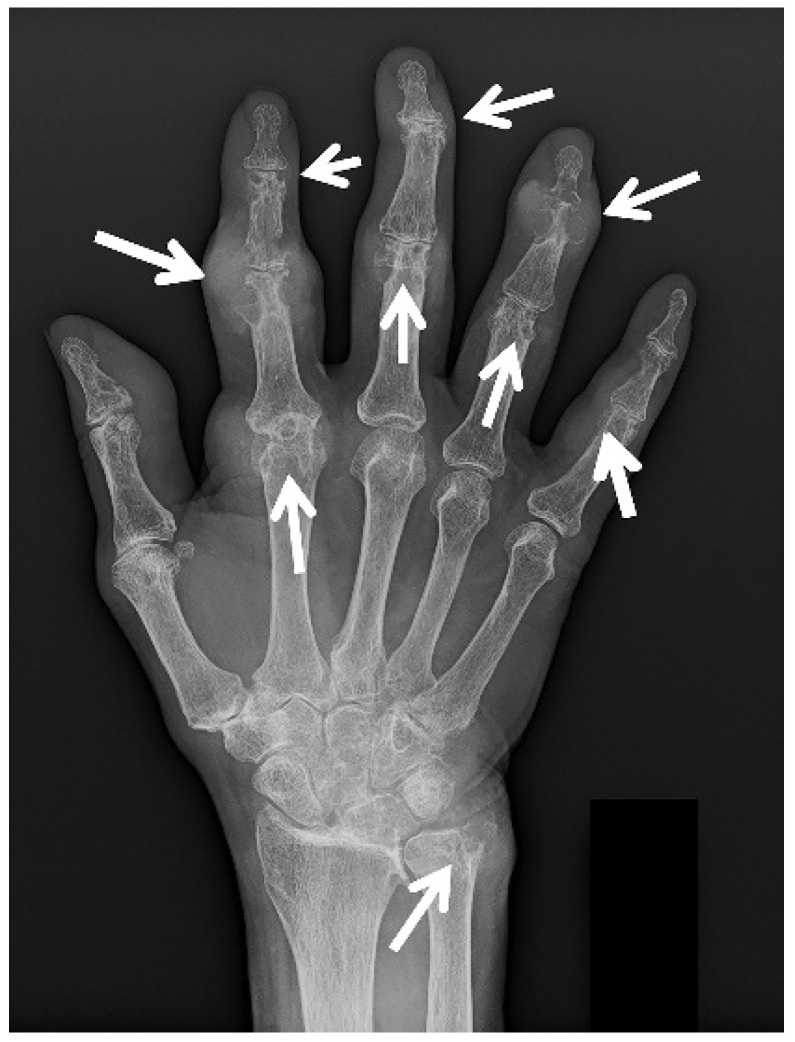
A 74-year-old man with gouty arthropathy involving bilateral hands. PA radiograph of the right hand shows erosive and cyst-like changes about multiple joints of the hand and ulnar styloid with adjacent dense soft-tissue nodules (arrows) consistent with gouty arthropathy. Several erosions have overhanging edges, most notable at the radial aspect of the index finger proximal interphalangeal joint. Note faint calcifications within the nodular thickening adjacent to the ulnar styloid erosion.

**Figure 3:**
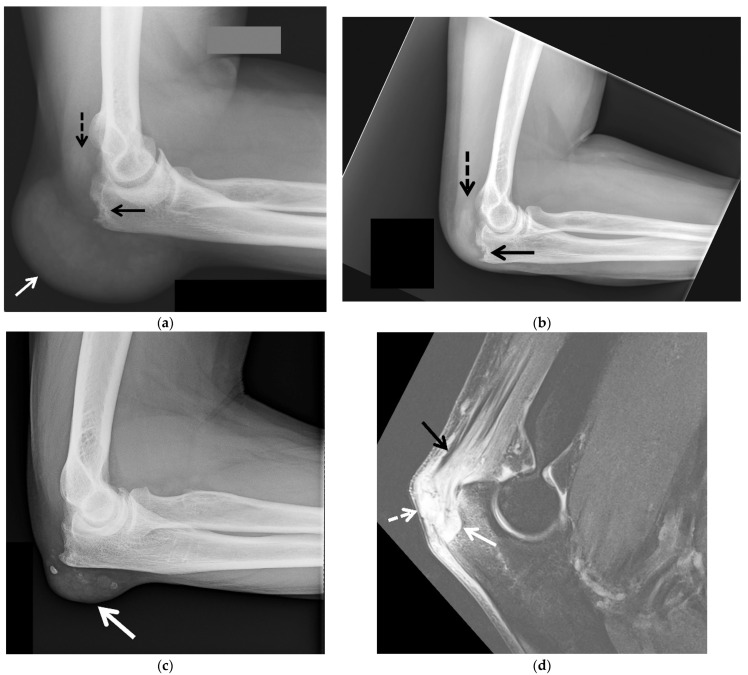
Tophaceous gout of the posterior elbow in 3 different patients. (**a**) Initial lateral radiograph of the left elbow in a 57-year-old man shows cortical erosion of the posterior olecranon (black arrow) with marked distension and somewhat increased density of the overlying olecranon bursa (white arrow). Note increased density of the distal triceps tendon (dashed black arrow). (**b**) Lateral radiograph of the left elbow of the same patient obtained after surgical debridement redemonstrated cortical erosion at the posterior olecranon (black arrow) and increased density of the distal triceps tendon (dashed black arrow) with interval marked improvement of posterior soft-tissue thickening. (**c**) Lateral radiograph of the right elbow in a 62-year-old man shows a soft-tissue mass involving the olecranon bursa with associated calcifications (arrow). (**d**) Sagittal T2-weighted with fat saturation MR image in a 60-year-old man shows a large bone erosion involving the posterior olecranon (white arrow) with associated mild bone marrow edema subjacent to a markedly thickened and irregular distal triceps tendon of heterogeneous increased signal intensity (black arrow). Note mildly distended irregular shaped, heterogeneous, predominantly high-signal-intensity olecranon bursa extending into the distal triceps tendon (white arrow) and additional high-signal-intensity subcutaneous edema at the posterior aspect of the elbow.

**Figure 4:**
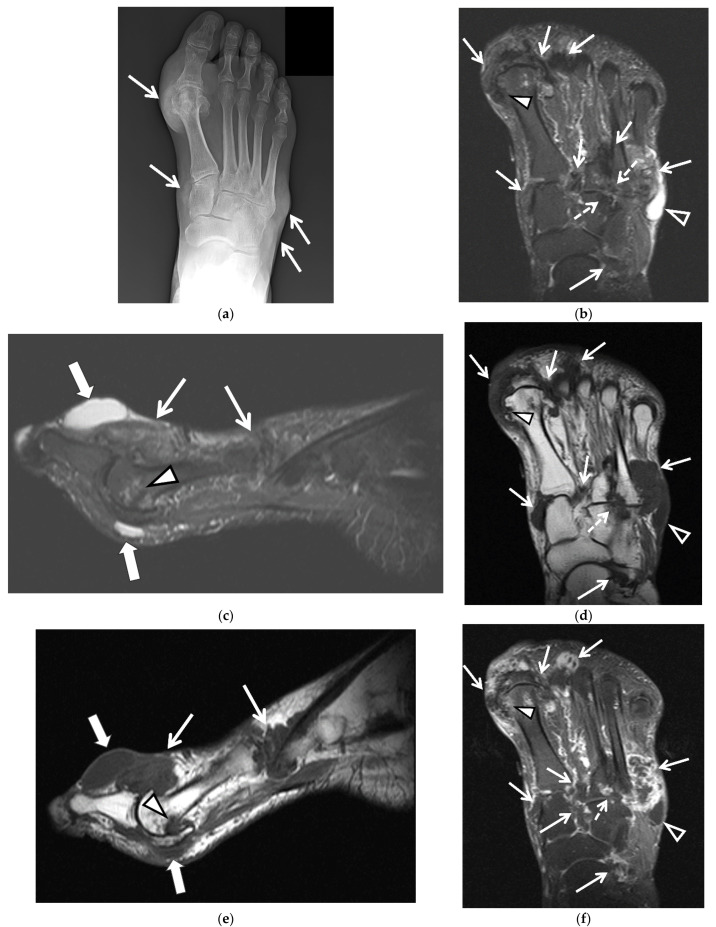

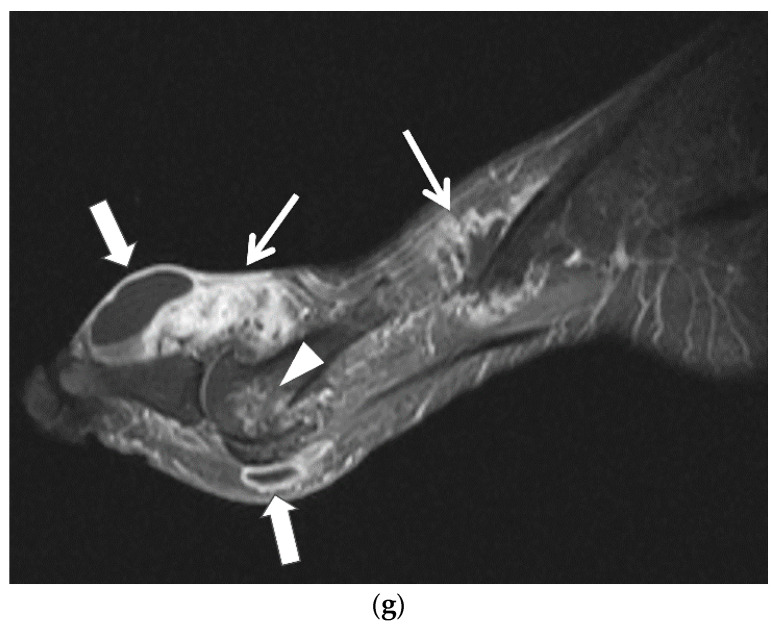
60-year-old man with tophaceous gout of the right foot. (**a**) AP radiograph of the right foot shows dense nodular soft-tissue thickening at the medial aspect of the first metatarsophalangeal and first tarsometatarsal joints and at the lateral aspect of the fifth tarsometatarsal joint (arrows). (**b**) Axial and (**c**) sagittal STIR and (**d**) axial and (**e**) sagittal T1-weighted MR images show multiple areas of intermediate-to-low signal intensity in the periarticular regions of the forefoot and midfoot related to MSU deposits and tophaceous gout (white arrows), cortical erosion at the medial aspect of the first metatarsal head (white arrowheads) and cortical erosions between the third and fourth tarsometatarsal joints (dashed white arrows) which show heterogeneous enhancement on the (**f**) axial and (**g**) sagittal T1-weighted with fat saturation post-contrast MR images. In (**b**) note high-signal-intensity lobulated adventitial bursal collection at the lateral aspect of the proximal fifth metatarsal bone (open white arrowheads) which shows intermediate-to-low signal in (**d**) and rim enhancement in (**f**). Additional adventitial bursae (white block arrows) are seen at the dorsal and plantar aspect of the first metatarsophalangeal joint, which show high signal in (**c**), intermediate-to-low signal in (**e**) and rim enhancement in (**g**).

**Figure 5:**
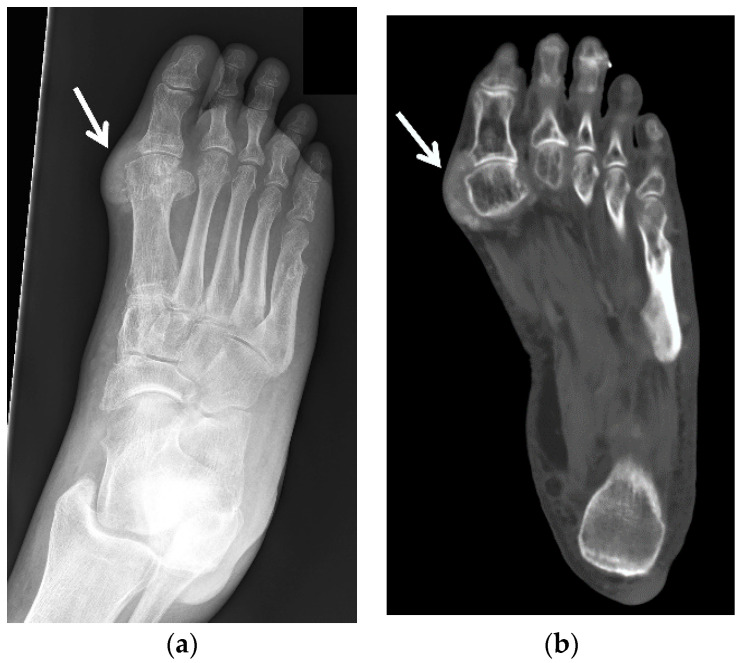
A 60-year-old man with gouty arthropathy of the right first metatarsophalangeal joint, also known as podagra. (**a**) Oblique radiograph and (**b**) axial CT image of the right foot shows erosive bone changes at the medial aspect of the great toe metatarsal head and proximal phalangeal base with overlying mass-like dense soft-tissue nodularity with faint calcifications consistent with MSU crystal deposition (arrows). Similar less pronounced findings are seen at the lateral aspect of the first metatarsophalangeal joint.

**Figure 6:**
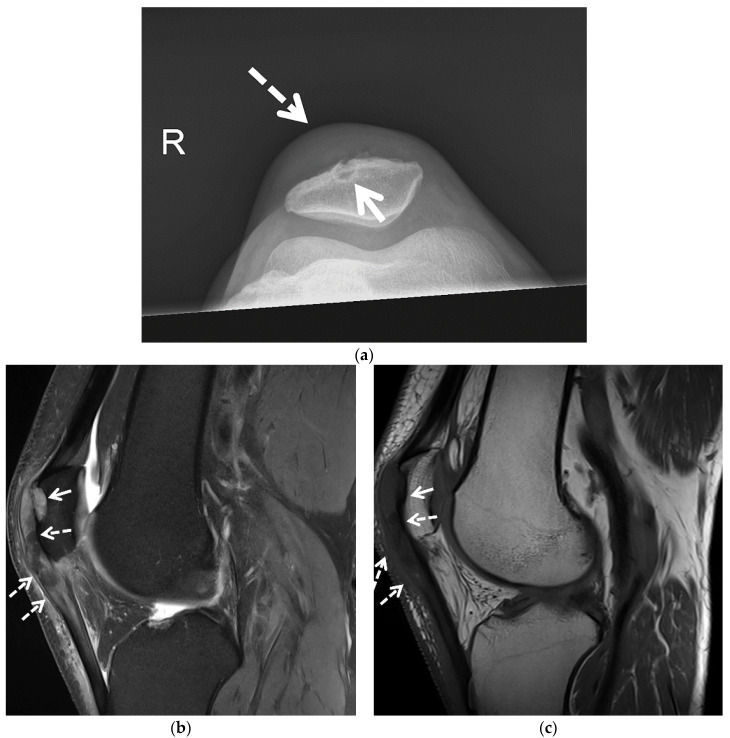
A 54-year-old man with tophaceous gout of the right knee. (**a**) Patellofemoral radiograph shows a well-marginated osseous erosion at the anterior aspect of the patella (arrow) with overlying soft-tissue edema (dashed arrow). Sagittal (**b**) proton density-weighted with fat saturation and (**c**) T1-weighted MR images show osseous erosion with soft-tissue deposit at the anterior surface of the patella (arrows) subjacent to the thickened heterogeneous quadriceps continuation in continuity with the heterogeneous thickened patellar tendon (dashed arrows). The affected extensor mechanism and the soft-tissue deposit at the anterior patellar osseous erosion site show heterogeneous increased signal in (**b**) and heterogeneous decreased signal in (**c**) consistent with MSU crystal deposition.

**Figure 7:**
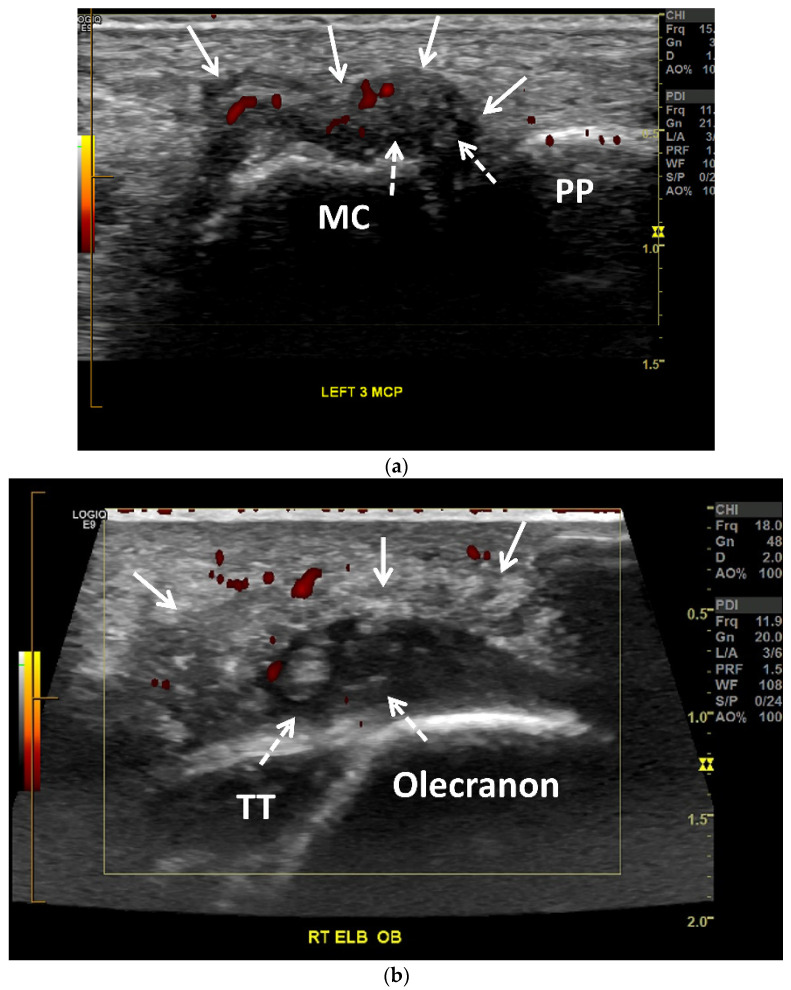
Gouty arthropathy in a 66-year-old woman. (**a**) Long-axis power Doppler US image along the dorsal aspect of the third metacarpophalangeal joint shows a moderate distension of the joint capsule (arrows) with numerous small echogenic foci related to MSU crystals (dashed arrows), creating “snowstorm” appearance. Note associated mild-to-moderate hyperemia (red). MC = metacarpal head. PP = proximal phalanx. (**b**) Obliquely oriented power Doppler US image along the posterior aspect of the right olecranon shows a heterogenous moderately distended olecranon bursa (arrows) with numerous small echogenic foci related to MSU crystals (dashed arrows). Note associated mild hyperemia (red). The distal triceps tendon (TT) is hypoechoic with scattered tiny hyperechoic foci consistent with tendinopathy and MSU crystal deposition.

**Figure 8:**
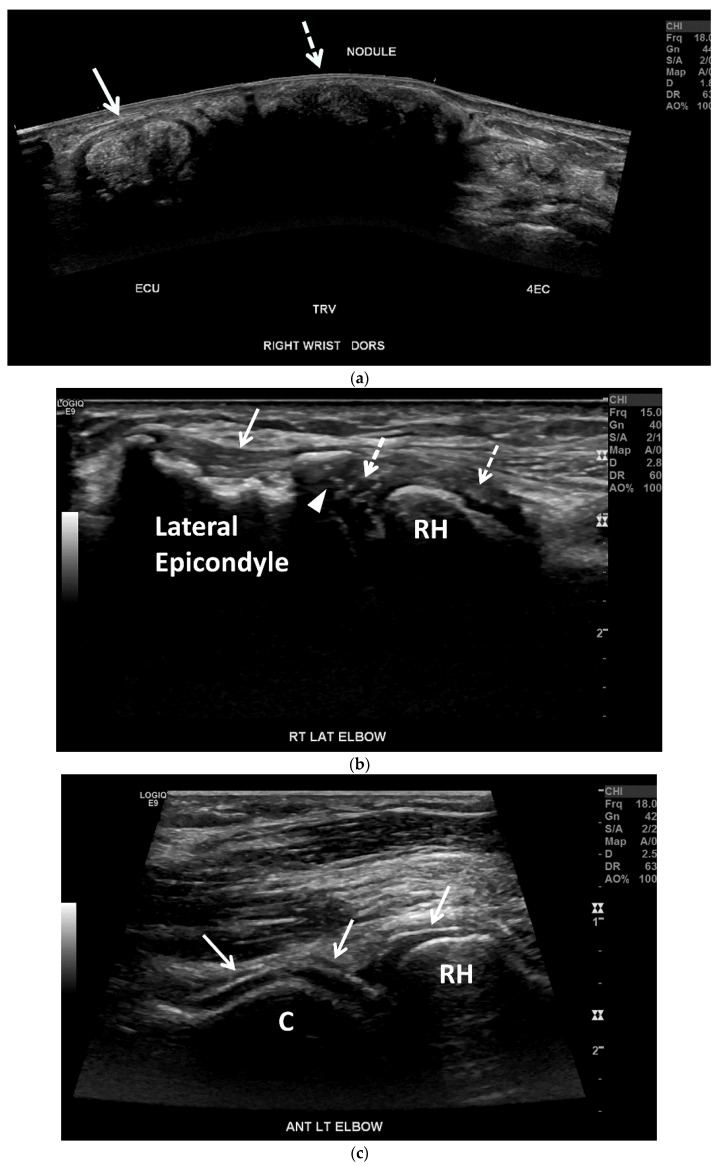
Tophaceous gout in a 73-year-old woman. (**a**) Panoramic transverse/short-axis gray-scale US image along the dorsal aspect of the right wrist shows markedly thickening and heterogeneous hyperechoic extensor carpi ulnaris tendon (ECU) related to tendinopathy with associated MSU crystal deposition (arrow). Note a large echogenic mass with posterior acoustic shadowing between the ECU and the fourth extensor compartment (4EC) related to a hard tophus (dashed arrow). (**b**) Long-axis gray-scale US image along the lateral aspect of the right elbow shows multiple small intra-articular echogenic foci related to MSU crystals (dashed arrows), undersurface erosion at the periphery of the capitellum (arrowhead) and cortical irregularity of the lateral humeral epicondyle subjacent to the heterogeneous common extensor tendon suggestive of chronic tendinopathy (arrow). C = capitellum. RH = radial head. (**c**) Long-axis gray-scale US image along the anterolateral aspect of the right elbow shows “double contour sign” along the radial head and capitellum articular cartilage related to MSU crystal deposition (arrows).

**Figure 9:**
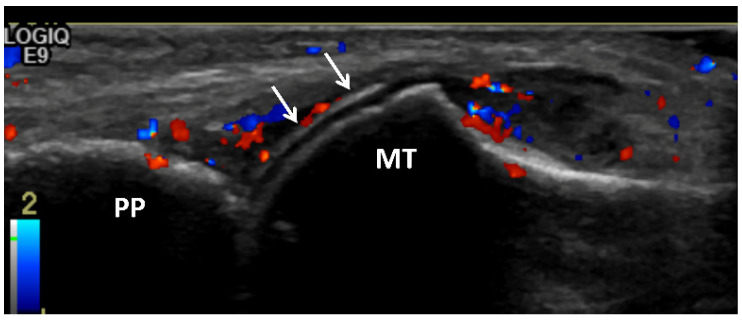
Gouty arthritis of the 1st metatarsophalangeal joint of 45-year-old male. Long-axis color Doppler US image along the dorsal aspect of the first metatarsophalangeal joint shows moderate distension of the joint capsule with moderate hyperemia consistent with synovitis. Note hyperechoic line that parallels the hyperechoic line of the subchondral bone, separated by anechoic cartilage along the metacarpal head (arrow) producing a “double contour” sign related to MSU crystal deposition. MT = metatarsal head. PP = proximal phalanx.

**Figure 10:**
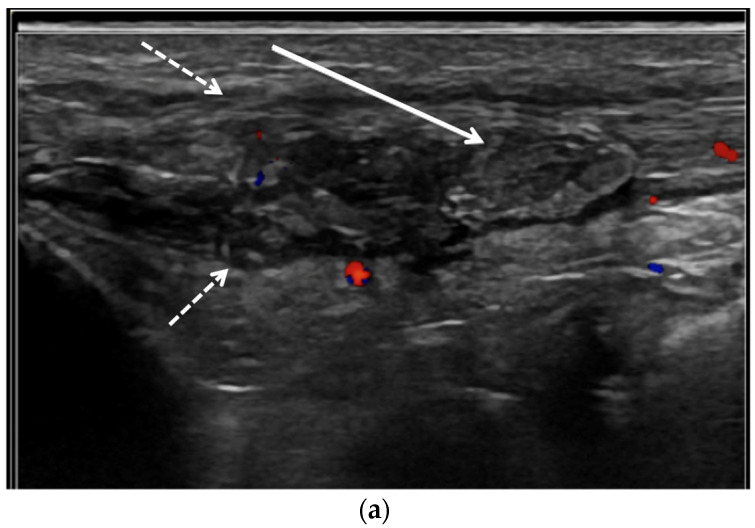

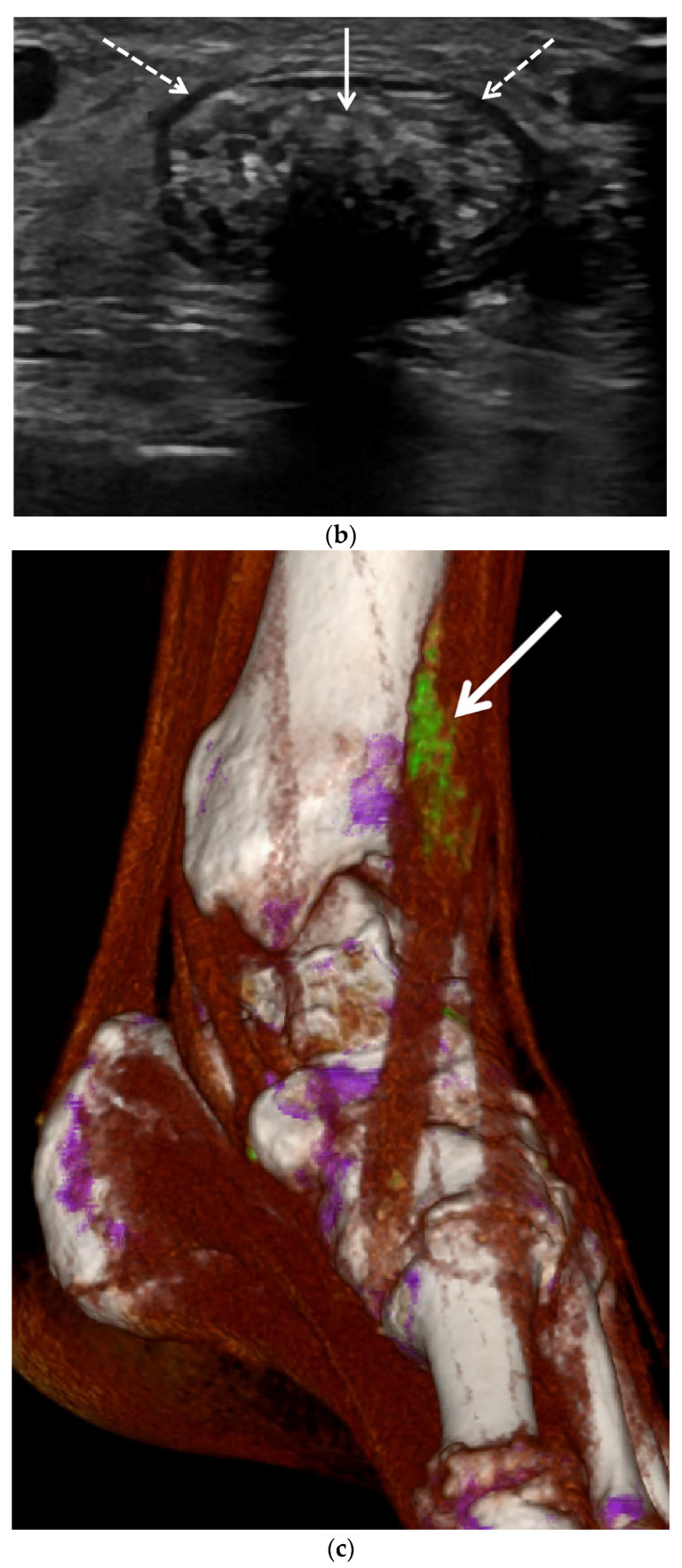
Gout of the tibialis anterior tendon. (**a**) Long-axis color Doppler US image along the dorsal aspect of the ankle and (**b**) short-axis gray scale US image of the tibialis anterior tendon in the same region show marked thickening and heterogeneous echogenicity of the tibialis anterior tendon consistent with severe tendinopathy and MSU crystal deposition (dashed arrows) with a more discrete echogenic focus of tophaceous gout (arrows) with posterior shadowing in (**b**). (**c**) A 3D reformatted dual energy CT (DECT) image of the ankle shows green encoded foci in the tibialis anterior tendon-related MSU crystal deposition concordant with US findings. DECT image acquired at 0.8–1.5 mm on a dual energy Siemens Somatom Force helical CT scanner using Syngovia post-processing software to demonstrate MSU crystals encoded in green.

**Figure 11:**
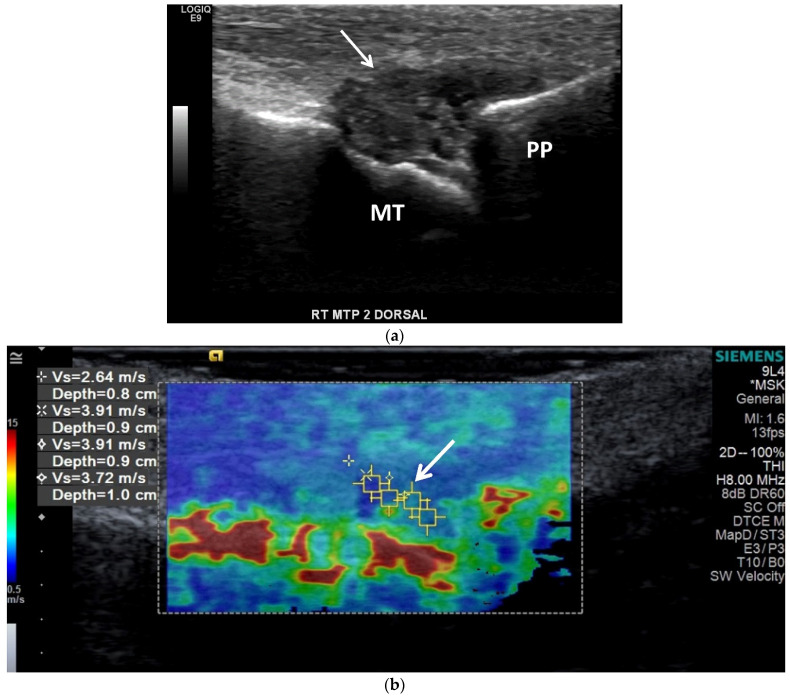
Tophaceous gout at the right second metatarsophalangeal joint in a 49-year-old man. (**a**) Long-axis gray-scale US image along the dorsal aspect of the second right metatarsophalangeal joint shows heterogeneous intra-articular gouty tophus (arrow) abutting the metatarsal head (MT). PP = proximal phalanx. (**b**) Color elastogram of the same region shows low shear-wave velocity (arrow) (mean, 3.54 m/s) consistent with a soft gouty tophus. SWE data were collected using an Acuson S3000 US scanner with an L9–4-MHz linear transducer.

**Figure 12:**
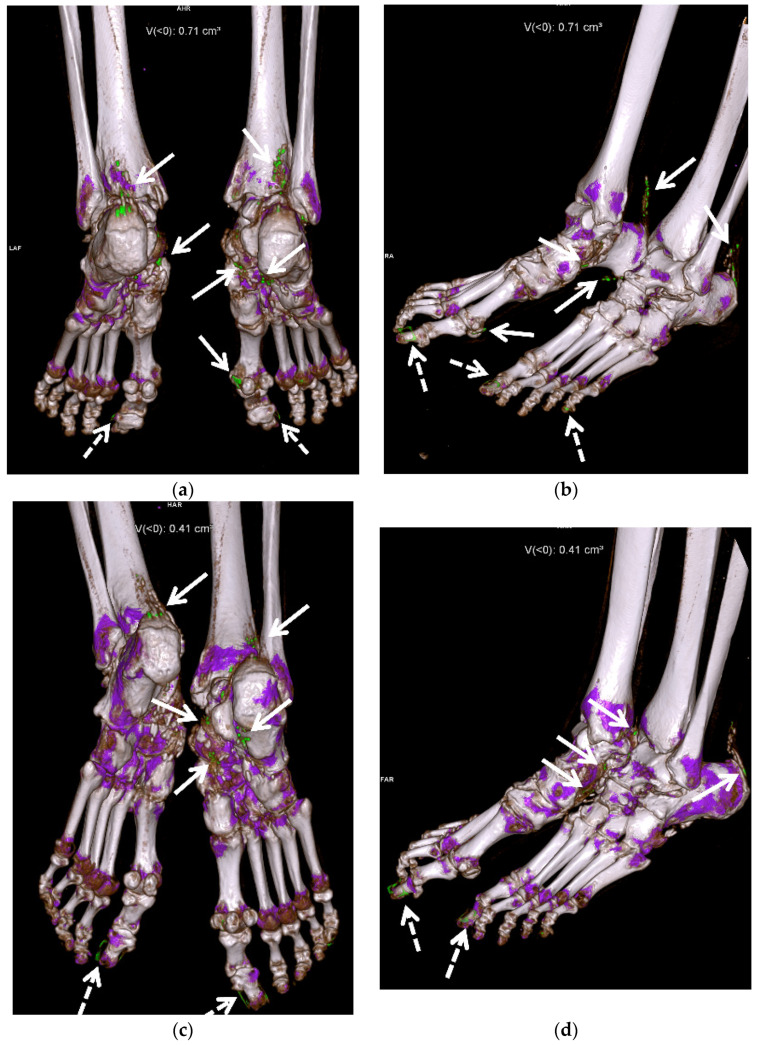
A 58-year-old female with extensive gouty arthropathy of the bilateral ankles and feet and decreased burden of MSU crystal deposition on the post-treatment 13-month follow-up DECT study. Pre-treatment (**a**,**b**) 3D reformatted DECT images of the bilateral ankles and feet show multiple green encoded foci of periarticular and articular MSU crystal deposition in both feet and distal Achilles tendons (arrows). Note green encoded foci about the great and little toenails related to imaging artifact (dashed arrows). (**c**,**d**) Three-dimensional reformatted DECT images of the bilateral ankles and feet obtained 13 months after initiation of treatment show interval decreased burden of periarticular and articular MSU crystal deposition in the same regions (arrows). Note green encoded foci about the great toenails related to imaging artifact (dashed arrows). All images acquired at 0.8–1.5 mm on a dual energy Siemens Somatom Force helical CT scanner using Syngovia post-processing software to demonstrate MSU crystals encoded in green.

**Figure 13:**
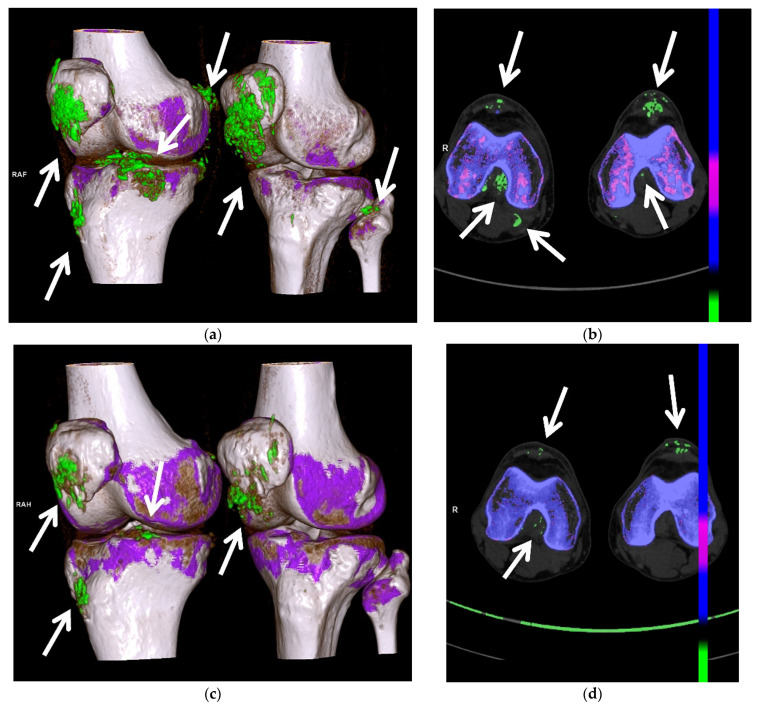
A 67-year-old male with extensive gouty arthropathy of both knees with decreased burden of MSU crystal deposition on the post-treatment 2-year follow-up DECT study. Pre-treatment (**a**) 3D and (**b**) 2D axial reformatted DECT images of the bilateral knees show multiple green encoded foci of extensive periarticular and articular MSU crystal deposition (arrows); (**c**) 3D and (**d**) 2D axial reformatted DECT images of the bilateral knees obtained 2 years after initiation of treatment show interval decreased burden of periarticular and articular MSU crystal deposition of both knees (arrows). All images acquired at 0.8–1.5 mm on a dual energy Siemens Somatom Force helical CT scanner using Syngovia post-processing software to demonstrate MSU crystals encoded in green.

**Figure 14:**
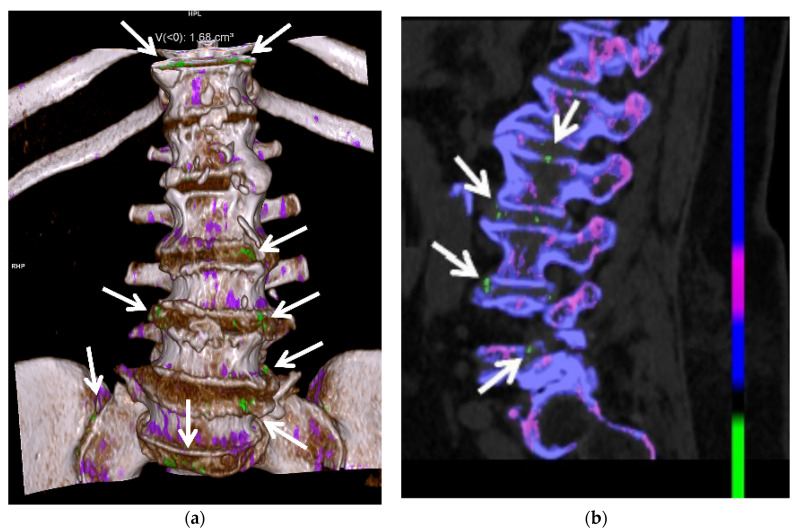
A 65-year-old female with spinal gout. (**a**) Three-dimensional and (**b**) two-dimensional sagittal reformatted DECT images of the lumbar spine show numerous green encoded foci of MSU crystal deposition along the lumbar and visualized lower thoracic spine, sacrum and sacroiliac joints. All images acquired at 0.8–1.5 mm on a dual energy Siemens Somatom Force helical CT scanner using Syngovia post-processing software to demonstrate MSU crystals encoded in green.

Imaging has an important role in the initial evaluation, differential diagnosis, and the treatment follow-up of gouty arthropathy. Imaging is also essential when the presentation is atypical or involves deep structures such as the spine, hip, or sacroiliac joint [15]. The imaging findings of gouty arthropathy on radiography, ultrasonography (US), computed tomography (CT) (both conventional and dual energy computed tomography (DECT)), and magnetic resonance imaging (MRI) (Figure 2, Figure 3, Figure 4, Figure 5, Figure 6, Figure 7, Figure 8, Figure 9, Figure 10, Figure 11, Figure 12, Figure 13 and Figure 14) are described, to include findings of the early, acute and chronic phases of gout. These findings include early MSU crystal deposits as well as later osseous erosions and tophi, which may involve periarticular tissues, tendons, and bursae.

Gout can be successfully treated with urate-lowering therapy, which involves often inexpensive, well-tolerated medications focusing on either reducing serum uric acid formation or increasing the renal excretion of uric acid [22,34]. Early diagnosis and treatment are essential to prevent the long-term sequelae of joint damage and tophus formation, and to prevent other comorbidities such as renal failure and cardiovascular disease [9,10,12,24,27,35,36]. Imaging is critical in monitoring response to therapy; drug therapy can be modulated based on imaging findings to optimize patient outcomes. This review article describes the current standard of care in imaging and treatment of gouty arthropathy.

## 2. Clinical

Risk factors for gout include hyperuricemia, diet, alcohol consumption, fructose consumption, medications, age, sex, genetics, acute illness, and several chronic diseases [1,7,16,23,24,35,36]. Diets high in purines, which are found in red meat and seafood, can exacerbate gout [7,35]. Consumption of alcohol, especially beer and hard liquor, less so wine, is associated with gout, including recurrent gout attacks [2,7,35,37]. Several medications, including certain diuretics, β-blockers, angiotensin-converting enzyme inhibitors, non-losartan angiotensin II antagonists, cyclosporine, tacrolimus, and low-dose aspirin have been linked to gout [1,24]. The incidence of gout is 2–6 times higher in men than in women [1,18]. Menopause is associated with an increased risk of gout, particularly in surgical menopause and early-onset natural menopause [38]. The risk of gout is increased in postmenopausal women who are not treated with hormone replacement therapy [1,38]. Additionally, while higher levels of serum uric acid levels increase the risk of gout among women in a graded manner, women have a lower risk of gout than men at the same uric acid level [39]. Approximately 80 percent of patients with gout have a positive family history of gout or hyperuricemia [6,7,15]. MSU crystal deposition can result in end-organ damage in the kidneys and heart [40]. Gout has been associated with diabetes, obesity, hyperlipidemia, metabolic syndrome, insulin resistance, hypertension, renal disease, stroke, neurodegenerative diseases, and cardiac disease, and can lead to premature death [1,7,13,17,22,23,35,39,41,42,43,44,45,46]. Gout is not associated with an increased risk of fractures [47].

There are four phases of gout: asymptomatic hyperuricemia, and acute, intercritical, and chronic gout. Acute gouty arthropathy is typically monoarticular and most commonly involves the lower limb, particularly the first metatarsophalangeal (MTP) joint, known as podagra, which is seen in greater than 50 percent of patients [4,13,14,16,17,18,19,22,23,36,48,49,50,51]. The hands, tarsal joints, knees, elbows, ankles, and bursae are other common sites of involvement [16,17,22,23]. MSU crystals also frequently deposited in and about the tendons and at the entheses [13,52].

In acute gout, patients present with rapid onset of severe pain, swelling, and erythema of the affected joint, tendon or bursa. An acute gout attack may mimic infection, but is self-limited, usually resolving within a few days or 1–2 weeks [1,23,48]. Serum uric acid may be normal during an acute attack of gout, whereas inflammatory blood parameters may be elevated [22]. Intercritical gout is the asymptomatic interval between episodes of acute gouty attacks.

Repetitive attacks of acute gout eventually lead to chronic arthropathy, with chronic synovitis, tophus formation and deposition, and finally, erosions and joint destruction [1,14,15,16,23,48]. If left untreated, about a third of patients will develop tophaceous gout within 5 years [17,53]. Tophi are non-tender soft-tissue masses found in the subcutaneous soft tissues, intra-articular or periarticular soft tissues, tendons, ligaments, retinacula, and bursae, secondary to chronic granulomatous reaction to MSU crystals [15,19,22,36,53,54,55]. They have a fibrovascular matrix with a center of MSU crystals surrounded by granulation tissue [36,56]. Tophaceous gout often occurs in the hands and wrists and along the extensor surface of the knees and elbows and may eventually result in osseous erosions [14,16,17,22]. Tophi tend to occur in areas of mechanical stress, such as adjacent to the first MTP joint, the Achilles and patellar tendons, and the olecranon and prepatellar bursae [36,56]. The cruciate ligaments, peroneal tendons, popliteus tendon, and infrapatellar fat pad are common sites of MSU deposition [6,7,21,52,55]. Tophi can also occur in the auricular appendages and the tip of the nose [21]. They result in cosmetic deformity and can cause impaired joint mobility [53,54].

Gout is often a clinical diagnosis. Patients with gout may have normal serum urate levels and hyperuricemia can be present in patients without gout. Aspiration of the affected joint or bursa is the gold standard for diagnosis. Joint aspirate is evaluated for crystals, as well as white blood cells to exclude infection. Macroscopically, MSU crystals are white in color [48]. They are needle shaped and have negative birefringence on polarized microscopy (Figure 1) [7,17,22,23,46,48,49,57]. The American College of Rheumatology (ACR) and European League Against Rheumatism (EULAR) created the 2015 Gout Classification Criteria, a useful diagnostic and classification algorithm for gout [3,58]. This algorithm utilizes clinical, laboratory, and imaging findings. In 2018, the EULAR provided updated evidence-based recommendations for the diagnosis of gout, recommending joint aspiration or tophus biopsy in every patient with suspected gout, and imaging in patients with atypical presentations when aspiration is not possible [8]. The differential diagnosis includes septic arthritis, acute calcium pyrophosphate arthropathy, reactive arthritis, and psoriatic arthritis. Chronic tophaceous gout may mimic rheumatoid arthritis, or rarely, tumor or other tumor like lesions [21]. Clinical history and laboratory evaluation are helpful to differentiate gouty arthropathy from infectious or other inflammatory arthropathies. Arthrocentesis and fluid evaluation remain the gold standard for the diagnosis of both gouty arthritis and septic arthritis. US has been shown to have a high specificity (greater than 90%) for the diagnosis of gout in patients with a symptomatic joint when compared to joint aspiration [59]. It should be remembered that septic arthritis and acute crystal arthritis can occur simultaneously. The presence of tophi on imaging suggests gouty arthropathy rather than an infectious or inflammatory arthropathy. Imaging, particularly DECT, can help differentiate acute calcium pyrophosphate arthropathy from gouty arthropathy, although a recent study showed that DECT may have a lower sensitivity for acute gout flares than previously described, and that DECT sensitivity for acute calcium pyrophosphate crystal arthritis is lower than that of US [60].

Although joint aspiration is the gold standard of diagnosis, aspiration is a mildly invasive procedure with complication risk, which may not be readily available and may be inaccurate in the setting of small volume joint effusion [5,9,24,46,50,57].

## 3. Imaging

### 3.1. Radiographs

Radiographs (Figure 2, Figure 3a–c, Figure 4a, Figure 5a and Figure 6a) are often the initial imaging modality in a patient suspected of having gout, as they are readily accessible and inexpensive. However, in early gout, radiographs are often normal or show only soft-tissue swelling. Erosions may not be apparent until 5–10 years after the initial acute gout attack [5,22,23,28,61,62]. The classic radiographic findings of longstanding gout include marginal and juxta-articular erosions (Figure 2, Figure 3a,b, Figure 5a and Figure 6a) with overhanging edges and sclerotic margins. Erosions may also be intra-articular. In chronic gout, tophi are seen as dense soft-tissue nodules (Figure 2, Figure 3a–c, Figure 4a, Figure 5a and Figure 6a) in the periarticular soft tissues or within the bursae with or without amorphous calcifications. Tophi may be radiographically occult if less than 5–10 mm [18,36]. Juxta-articular erosions are usually seen adjacent to tophi, as they frequently represent intraosseous extension of tophi [18]. Joint spaces and periarticular bone density are maintained until late disease. Radiography has a reported sensitivity of 31% and specificity of 93% in the diagnosis of gout [19,28].

### 3.2. Ultrasound (US)

Diagnostic US (Figure 7, Figure 8, Figure 9, Figure 10a,b and Figure 11), utilizing high frequency linear transducers (12 MHz and higher) is useful in the evaluation of gout. US can provide a diagnosis of gout, can be used to guide joint aspiration or soft-tissue biopsy, and can be used to monitor response to therapy. US has high spatial resolution and is multiplanar, uses no ionizing radiation, is a dynamic examination, is widely available, is relatively low cost, and is portable. However, US is operator-dependent, with a steep learning curve, and is limited to more superficial joints. US findings include joint effusion, synovitis, MSU crystal deposition, tophi and erosions [7,17]. A meta-analysis by Lee et al. in 2018 showed that US has an overall specificity of 89% and sensitivity of 65.1% for the diagnosis of gout [57].

The Outcome Measures in Rheumatology Clinical Trials (OMERACT) task force subgroup created consensus-based definitions on the US elementary gout lesions of double contour, aggregates, tophi, and erosions [63]. OMERACT defined aggregates as “heterogeneous hyperechoic foci that maintain their high degree of reflectivity even when the gain setting is minimized or the insonation angle is changed and which occasionally may generate posterior acoustic shadow” [63]. A new consensus definition was created in 2021, defining aggregates as “bright hyperechoic, isolated spots too small to fulfil the tophus definition and characterized by maintaining their high degree of reflectivity when the insonation angle is changed” [64].

Joint effusions in gout may range from simple, anechoic fluid to heterogeneous hyperechoic collection with synovitis (Figure 7a, Figure 8b, Figure 9, Figure 10a,b and Figure 11a) [17,28,65]. MSU crystal aggregates/microtophi can be seen in the joint as floating echogenic foci, known as the “snowstorm appearance” (Figure 7a) [7,9,13,15,17,18,28,29,31,36,59,65,66,67]. These echogenic foci could represent joint bodies, and US cannot differentiate MSU crystals from calcifications; DECT can be utilized for this [13]. Synovitis occurring in gout is usually heterogeneously hyperechoic due to MSU crystal deposition [7,15,18]. Intrinsic hyperechoic streaks and a hypoechoic peripheral rim with increased vascularity are also often present within the synovium in gout [7,28]. Synovial hyperemia, visualized on color or power Doppler imaging, may be secondary to active inflammation or may be due to the fibrovascular matrix of tophi, and may also be present in subclinical disease (Figure 7 and Figure 9) [18,36].

MSU crystals also precipitate on the superficial layer of the hyaline cartilage, producing an irregular hyperechoic line over the anechoic cartilage. This hyperechoic line parallels the hyperechoic line of the subchondral bone, separated by anechoic cartilage, producing the “double contour sign” (Figure 8c and Figure 9) [7,9,13,15,17,18,29,31,36,46,57,59,65,66,67]. OMERACT defined the double contour sign as “abnormal hyperechoic band over the superficial margin of the articular hyaline cartilage, independent of the angle of insonation and which may be either irregular or regular, continuous or intermittent and can be distinguished from the cartilage interface sign” [63]. The sensitivity of the double contour sign in patients with gout ranges from 25–95% [7,17,25,66,67,68,69]. The double contour sign should not be confused with hyperechoic foci within the cartilage, as in calcium pyrophosphate deposition, or with the normal cartilage interface sign [7,13,15,29,65,66].

In chronic gout, erosions (Figure 8b) can be visible on US [7,9,13,17,18,31,70]. OMERACT defined erosions as “an intra- and/or extraarticular discontinuity of the bone surface (visible in 2 perpendicular planes)” [63]. Post-traumatic changes, degenerative changes, and normal variants can mimic erosions. Adjacent tophi, synovitis, and hyperemia with color Doppler imaging suggest active erosions [7]. US has been shown to detect erosions in gout earlier and at smaller size than radiography [15,17,25,26,66]. However, US can underestimate the extent of erosions compared to MRI [15,71].

OMERACT defined tophi as “a circumscribed, inhomogeneous, hyperechoic and/or hypoechoic aggregation (that may or may not generate posterior acoustic shadow) which may be surrounded by a small anechoic rim” [63]. On US, tophaceous deposits (Figure 8a and Figure 10a,b) in chronic gout have a hyperechoic center, representing the MSU crystals, with an anechoic rim, representing the granulation tissue, and may have either a nodular or an infiltrative appearance (“soft tophi” (Figure 11)), or posterior acoustic shadowing (“hard tophi” (Figure 8a and Figure 10b)) [7,15,17,18,21,29,36,65,66,67]. Tophaceous deposits within tendons are usually hypoechoic with scattered hyperechoic foci (Figure 7b), resulting in disruption of the normal fibrillar tendon echotexture; if chronic, hyperechoic bands with posterior shadowing are often present (Figure 8a and Figure 10a,b) [17,65].

Shear wave elastography (SWE) (Figure 11b) uses ultrasound to obtain quantitative measurements of tissue elasticity to assess intrinsic tissue stiffness [72]. Forced acoustic radiation force from a linear US array generate shear waves which propagate perpendicularly to the primary US wave to produce local tissue displacement. Displacement and velocity are tracked as the shear waves propagate, and the tissue displacements are used to calculate shear wave velocity and shear modulus [72]. Quantitative shear modulus maps are produced to show shear wave velocities (meters per second) and tissue elasticity (kilopascals) [72]. SWE has been shown to be able to quantitatively differentiate gouty arthropathy from non-gouty arthropathy in patients without acute gout [73]. Wang et al. showed that the stiffness of the synovium is higher in the intercritical phase of gout than in the acute phase, and thus increases diagnostic performance in differentiating acute from intercritical gout in comparison with conventional US [74]. In gouty tophi, shear wave velocities are dependent on consistency, with harder tophi having higher velocities than soft tophi [72].

### 3.3. Computed Tomography (CT): Conventional and Dual Energy (DECT)

Both conventional CT (Figure 5b) and DECT (Figure 10c, Figure 12, Figure 13 and Figure 14) are useful in the evaluation of gout. CT has high spatial resolution, is multi-planar and can visualize deep structures. This imaging modality is limited by cost, use of ionizing radiation, and lack of portability. Conventional CT is more widely available than DECT.

Conventional CT can be used to detect erosions and tophi in chronic gout [15]. Tophaceous nodules have a density of approximately 170 Houndsfield units [15]. Hyperdense deposits can be seen in the joints in acute gout (Figure 5b) [51].

DECT utilizes the photon-energy-dependent attenuation of different materials to identify MSU crystals. It uses two different energies (80 and 140 kVp) to determine the composition of materials using the properties of differing atomic number and mass density, which can be color coded during post-processing, to differentiate urate acid crystals/tophi from other calcifications [7,10,11,12,13,14,15,18,31,36,46,49,62,75,76]. DECT directly images MSU crystal deposition, and thus is independent of the current serum urate level [14]. DECT is a part of the 2015 and 2018 ACR/EULAR classification criteria for gout [3,8,76]. Quantitative measurement of tophi can be obtained with DECT [10,12,13,18,31,36,46,49,75]. Peripheral limbs may be scanned to create urate maps (Figure 12 and Figure 13) [12,50,75]. Subclinical disease can also be detected with DECT, often within the joints and tendons, without tophi [14,36,46,77].

DECT is both sensitive (78–100%) and specific (89–100%) in identifying MSU deposition [5,10,11,12,14,31,50,75]. The sensitivity of DECT is lower for acute gout than for chronic gout [76,77]. In early gout, false-negative imaging can result if the MSU volume is low or the tophi are very small (less than 2 mm) [7,18,31,62]. Lee et al. showed that sensitivity of DECT for early gout can be increased when combined with conventional CT [51]. In this study, conventional CT was used to evaluate for the presence of hyperdense deposits in patients with suspected early gout. These deposits are nonspecific, and could represent MSU crystals or other crystal arthropathies such as calcium pyrophosphate deposition disease, which can be differentiated by DECT.

DECT is useful in patients with atypical clinical presentations and unusual site of involvement [14,49,78]. Gout in the axial skeleton (Figure 14) can be a challenging diagnosis due to rarity of presentation and difficulty of obtaining tissue sampling, and DECT can guide diagnosis when gout is suspected [24,79]. Zhu et al. showed that DECT is more accurate in the diagnosis of gout than US in the joints of the upper limb, thought to be due to the complex anatomy and smaller size of the upper-limb joints compared to the lower-limb joints [40]. Klauser et al. also showed that the percentage of gouty deposits detected by US was significantly lower than that by DECT, particularly within the extra-articular spaces [30]. DECT may be very useful when joint aspiration is either non-feasible or non-diagnostic.

Artifacts are prevalent in DECT [7]. These artifacts include green pixels in the skin particularly the heels, and in the nails and nail beds (Figure 12), due to keratin in callous and thickened nails, and artifactual pixilation from motion and metal [7,10,18,24,36,46,76]. False-positive imaging can occur in areas of apposed skin [75]. It is uncertain if urate-like pixilation uptake in vasculature represents true MSU crystal deposition or artifact, and it is under further investigation for the cardiovascular system [24,45,76]. DECT has lower specificity for gout in osteoarthritic knees [11,18,31].

A recent study shows promising results in the use of DECT to detect cardiovascular MSU deposits in the coronary arteries and the aorta of gout patients compared to controls [45]. MSU crystal detection on DECT has been shown to be predictive of developing new cardiometabolic disease and for increased mortality [80].

### 3.4. Magnetic Resonance Imaging (MRI)

MRI (Figure 3d and Figure 4b–g) has high-contrast and spatial resolution, permitting detailed evaluation of the bone marrow, periarticular soft tissues, and articular cartilage, without the use of ionizing radiation. Limitations of MRI include long examination times, high cost, limited availability, lack of portability, claustrophobia, and restrictions by some implanted medical devices.

MRI is useful in evaluating gout in the spine and other deep areas not amenable to clinical or US evaluation [15]. In early gout, MRI can show bone-marrow and soft-tissue edema as well as simple or complex joint effusions and synovitis [18]. MSU crystal deposition on the surfaces of the hyaline cartilage is not visible by MRI [15].

On MRI, erosions (Figure 3d and Figure 4b–g) have cortical disruption with overhanging edges with associated intra-articular and extra-articular soft-tissue tophi that may calcify. Acute erosions will have irregular margins with adjacent enhancing active synovitis while chronic erosions will appear more well-marginated with cortication, often without adjacent active synovitis [17]. Minimal bone marrow edema is present around erosions until late disease, and cartilage surfaces are spared from erosive changes until late disease [18,36,81]. The presence of tophi predicts eventual erosions, but the presence of bone marrow edema and synovitis do not [18,82].

MRI can demonstrate the extent of tophi, including within the bursae and tendons (Figure 3d and Figure 4b–g) [15]. Tophi show intermediate-to-low signal on T1-weighted (T1W) MR images and heterogeneously high-to-intermediate signal on fluid-sensitive images, with heterogenous enhancement following intravenous administration of gadolinium-based contrast (Figure 3d and Figure 4b–g) [7,15,18,31,36,55]. Areas of low signal and non-enhancement suggest the presence of calcifications, especially if small can be missed on MR images.

### 3.5. Nuclear Medicine

Nuclear medicine imaging studies are not the preferred modalities for evaluation of gouty arthropathy. However, as gouty arthropathy is common, it may be encountered on these studies. On bone scintigraphy, gout can manifest as articular, periarticular, and soft-tissue radiotracer uptake [83,84]. Gouty tophus has been shown to be moderately hypermetabolic on fluorodeoxyglucose positron emission tomography CT (FDG PET CT) [85,86,87]. Tophaceous gout in the spine will show uptake on Gallium-67 imaging [88].

## 4. Laboratory Evaluation

Blood tests may show elevation of the erythrocyte sedimentation rate (ESR) or C-reactive protein (CRP) during a gout flare. These markers of inflammation can be elevated due to other diseases that can cause inflammatory arthritis, however. Serum urate levels may be elevated but can also be low or normal during a flare [89]. An elevated serum urate level may suggest gout but is not enough to make a diagnosis.

Analyzing an aspirated synovial fluid, on the other hand, can confirm a gout diagnosis. The synovial fluid in gout is typically inflammatory in nature, with a white blood cell count of at least 2000 per mm^3^. A flare is characterized by the presence of MSU crystals in synovial fluid found by examination of the fluid using compensated polarized light microscopy. MSU crystals are negatively birefringent and are needle shaped. Sensitivity of laboratory crystal analysis for MSU crystals ranges from 63–78%, and specificity ranges from 93–100% [90].

## 5. Medical Management of Acute Gouty Arthritis

The primary goal during an acute gout flare is quick and safe termination of pain. While a gout flare may resolve untreated within days or weeks, symptoms may resolve more quickly with the use of various different treatments.

### 5.1. Glucocorticoids

Oral glucocorticoids are often used in patients with a typical gout flare who are able to take oral medications but have contraindications to the use of nonsteroidal anti-inflammatory drugs (NSAIDs). A typical regimen would be prednisone initiated at 30–40 mg per day. This is then tapered over 7–10 days, but duration of the taper may be needed for up to 21 days. Glucocorticoids are similar (or even better) in efficacy and have no greater risk of adverse effects compared to other agents used to treat acute gout [91,92,93]. However, other treatment options may be preferred among those with suspected infection, prior glucocorticoid intolerance, or uncontrolled diabetes mellitus. Common adverse effects of glucocorticoid use include mood changes, hyperglycemia, hypertension, and fluid retention.

Intra-articular glucocorticoid injection may be an indication among those who are unable to take oral medications and with one or two active inflamed joints. Typically, triamcinolone acetonide (up to 40 mg for a large joint and 20 mg for medium joint) or methylprednisolone acetate is used. While the evidence for its use in the treatment of gout flares is limited, it can be a relatively safe and efficacious treatment [94]. In addition, parenteral glucocorticoids may be indicated among those who cannot take medications orally and are not candidates for intra-articular therapy (e.g., with >2 active inflamed joints) [95]. Intravenous methylprednisolone (20 mg) may be helpful among those with polyarticular involvement, with an intravenous access, and with no contraindication to glucocorticoids. Intramuscular triamcinolone acetate treatment (40–60 mg) may be an alternative treatment for patients with similar conditions.

### 5.2. Nonsteroidal Anti-Inflammatory Drugs (NSAIDs)

NSAIDs are very good alternatives to oral glucocorticoids in the treatment of acute gout [96,97,98,99]. They are particularly appropriate among younger patients who do not have renal, cardiovascular, or active gastrointestinal disease. Naproxen (500 mg twice daily) or indomethacin (50 mg three times a day) are traditionally used. However, other NSAIDs such as ibuprofen (800 mg three times daily), diclofenac (50 mg two–three times daily), celecoxib (100 mg twice daily), and meloxicam (15 mg daily) are probably as efficacious [96,97,98]. NSAIDs work best when initiated within 48 h of symptom onset and can be discontinued two to three days after clinical symptoms have resolved. There are contraindications to NSAID use, however, including: chronic kidney disease (typically creatinine clearance < 60 mL/min), active gastrointestinal ulcer, cardiovascular disease (especially heart failure), or concomitant treatment with anticoagulants. Adverse effects from short-term use of NSAIDs are rare but include gastrointestinal disturbances and worsening renal function.

### 5.3. Colchicine

Low-dose oral colchicine can be used for acute gouty flare, especially among patients with intolerance or contraindications to glucocorticoid and NSAID use. A typical dosage is a total of 1.8 mg during the first day of therapy, and treatment is indicated for the duration of the flare at 0.6 mg once or twice daily [100]. It works best when taken at the initial onset of gout symptoms. Common adverse effects include diarrhea and abdominal cramping, especially with high dose therapy. Colchicine would be contraindicated among those with significant renal or hepatic insufficiency, and among those taking medications that may inhibit the cytochrome P450 system component CYP3A4 (e.g., HIV protease inhibitors, azole antifungals) or medications that inhibit the P-gp efflux pump (e.g., macrolide antibiotics, tacrolimus, cyclosporine) [100,101]. Severe side effects, including blood cytopenias, myopathy, and peripheral neuropathy, are relatively rare.

### 5.4. Interleukin-1 (IL-1) Inhibitors

While IL-1 inhibitors may benefit certain patients with an acute gouty attack, they are typically reserved for those for whom other available treatments have failed or who have contraindications to them [91,102,103]. Anakinra (100 mg daily) is the preferred IL-1 inhibitor treatment for acute gout due to its short half-life and its relatively modest cost compared to other IL-inhibitors. It is given subcutaneously daily until symptoms of the gout flare improve, and can be useful among hospitalized patients with an active infection or who are in the perioperative setting [102]. Recurrent flares are not uncommon among anakinra-treated patients, however.

### 5.5. Urate-Lowering Therapies

Lifestyle modifications that may decrease patient urate levels include weight reduction, cessation of excessive alcohol consumptions, and moderation in the consumption of purine-rich food. However, lifestyle modifications may not be adequate, and pharmacologic therapies may be indicated in patients with chronic gout. Specific indications for the initiation of pharmacologic urate-lowering therapies in gout are as follows: (1) frequent (≥2 annually) gout flares; (2) evidence of radiographic damage due to gout; and (3) presence of ≥1 subcutaneous tophi [104]. Upon initiation of urate-lowering therapy, patients also receive prophylactic treatment to decrease and prevent recurrent gout flares. Prophylactic treatment options include colchicine, a NSAID, or a low-dose glucocorticoid [104,105]. Achieving a serum urate level of <6 mg/dL is the recommended goal according to the 2020 ACR Guideline for the Management of Gout [104].

### 5.6. Allopurinol

Allopurinol, a xanthine oxidase inhibitor, is the first-line urate-lowering therapy for most patients [104]. The starting dose is typically ≤100 mg/day with dose increase by 100 mg every two to four weeks to reach and maintain the target serum urate level. Patients of Southeast Asian descent (e.g., Chinese, Korean, Thai) and African-Americans should be tested for the HLA-B*5801 allele; HLA-B*5801 positive individuals have a much higher risk of developing severe cutaneous adverse reactions (SCARs) [106]. For a similar reason, patients with moderate-to-severe chronic kidney disease (stage ≥ 3) can be started on a lower dose. Doses up to 300 mg/day and even higher are often used. Mild adverse effects include rash, leukopenia or thrombocytopenia, and diarrhea. Severe reactions, which are rare, include DRESS (drug reaction with eosinophilia and systemic symptoms) syndrome and SCARs.

### 5.7. Febuxostat

Febuxostat is an alternative xanthine oxidase inhibitor that can also be used for treatment of hyperuricemia. A daily dosage (40 mg or 80 mg) produces a reduction that is equivalent or better than that seen in patients treated with allopurinol 300 mg once daily [107,108]. It can be given safely to those with renal insufficiency, but the cost of treatment tends to be higher compared to allopurinol. Potential adverse effects include transaminitis, nausea, arthralgia, and rash. Of particular concern, febuxostat, compared to allopurinol, is associated with higher risk of cardiovascular mortality and all-cause mortality. The medication currently carries a boxed warning for increased risk of death [109].

### 5.8. Probenecid

Probenecid is the only uricosuric medication that has been approved by the United States Food and Drug Administration for the purpose of promoting renal uric acid clearance. It is used uncommonly in the United States, however, as it is only appropriate for gout patients with relative renal underexcretion of uric acid. Multiple daily dosing (250–1000 mg) is also required, and the medication is not effective among those with moderate-to-severe kidney disease [110]. Potential side effects include gastrointestinal intolerance, rash, and kidney stones. A combination xanthine oxidase inhibitor and probenecid, however, can be effective when monotherapy with an oral urate-lowering drug fails [110].

### 5.9. Pegloticase

A recombinant form of the enzyme uricase, pegloticase can cause a rapid reduction of serum urate level. However, due to its cost and potential adverse effects, the ACR recommends against its use as first-line therapy for gout [104]. It is administered intravenously every two weeks (8 mg), and is associated with more rapid reduction of gout signs and symptoms compared to other urate-lowering therapies [111]. However, its efficacy and safety are influenced by the development of antidrug antibodies, which are associated with a rise in serum urate levels and the appearance of infusion reactions [112]. Hence, the medication is often reserved for patients with advanced gout and when other urate-lowering therapies are ineffective or contraindicated.

## 6. Surgical Management of Chronic Gouty Arthritis

Despite medical treatment, some patients may eventually need surgical intervention. Common indications for surgical intervention include restoration of function, treatment of symptoms such as pain and infection, and restoration of cosmesis, particularly of the hand and wrist [113]. Dissection and curettage can be used for infiltrative lesions of tendons [113]. In advanced disease, resection of the affected tendon with either primary repair or tendon transfer may be necessary [113]. Other surgical interventions include tenosynovectomy, tophectomy, hydrosurgery, and arthrodesis [113]. In the MTP joints, several surgical options are available, including both joint-sparing and joint-destructive procedures [114]. Surgical interventions in patients with advanced disease have high morbidity. These patients are at high risk of delayed postoperative wound healing due to poor circulation to the overlying skin and potential leakage of inadequately removed urate deposits through the skin [113]. Some surgeons endorse earlier debulking surgery to decrease involvement of vital structures, decrease risk of infected or ulcerated tophi, and thus decrease morbidity and increase the ability to restore function [113].

## 7. New Horizons in the Treatment of Gouty Arthritis

Recent research on the anti-inflammatory effect of electromagnetic fields (currently used to promote bone healing) to decrease chronic inflammation and synovitis and thus prevent the progression of joint destruction appears promising [115,116,117].

## 8. Imaging of Treatment Response

The OMERACT working group established guidelines in 2015 for research on the role of imaging in gout therapy to include MSU deposition, joint inflammation, and bone erosion [32,118]. OMERACT endorses the following chronic gout domains: serum urate, tophus, pain, flares, and patient global assessment in assessing remission [119,120].

Radiographs can be used to monitor tophus size as an indicator for response to therapy [19,61].

US may be used to monitor therapy. With successful therapy, the double-contour cartilage sign can resolve, and tophi can reduce in size [7,9,20,26,27,31,32,67,118,121,122].

US can show resolution of urate deposits in patients on urate-lowering therapy with greater decrease of US tophus size and resolution of double-contour cartilage sign occurring in patients with lower serum urate levels than in those patients with higher serum urate levels [123]. It has been shown that the MSU crystal burden on US can predict fulfilling remission criteria for gout: the lower the baseline MSU burden estimated by US, the higher the chance to fulfil the remission criteria at 12 months, with the double-contour cartilage sign being the most useful measure [124]. Christiansen et al. showed that US can detect decreases in urate crystal deposition (double contour sign, tophi, aggregates and erosions) in patients on successful urate-lowering therapy [125]. Hammer et al. showed that patients treated with urate-lowering therapy to attain target serum urate levels (treat-to-target) had reduction of crystal deposition, with decreased double contour sign, decreased tophi, and decreased MSU aggregates on US [126]. Ebstein et al. found that a high reduction in US tophus size is associated with lower probability of relapse following the cessation of gout prophylaxis therapy [127].

DECT provides accurate and reproducible quantification of MSU crystal deposits and can be used to evaluate change in size and burden of tophi in response to therapy and provide quantitative measurement of response to therapy (Figure 10c, Figure 12, Figure 13 and Figure 14) [7,14,31,32,54,62,118]. Greater number and volume of MSU crystal depositions on DECT correlate with greater disease severity, but even patients with controlled gout (target serum uric acid levels and no palpable tophi) can have crystal deposition on DECT, suggesting the need for increased urate-lowering therapy [128]. Dalbeth et al. showed that in patients with gout treated with allopurinol, remission as defined by these criteria (with the exception of flares and pain) is associated with less DECT urate crystal deposition [119]. The Gout in Norway study (NOR-Gout) showed that patients with gout treated to target with urate-lowering therapy had sustained reductions in urate deposition on DECT [129]. Dalbeth et al. also showed that treating to target led to decreased erosion scores and decreased urate deposition on DECT [130].

MRI can detect change in size of tophi as well as resolution of bone marrow edema [15,31]. US and MRI can detect resolving joint effusions and synovitis [15].

## 9. Conclusions

Imaging shows both the soft-tissue and osseous changes that occur in gout secondary to inflammatory changes from the deposition of MSU crystals. Current EULAR recommendations state that US is helpful in establishing a diagnosis of gout by detection of either a double contour sign on cartilage surfaces or non-clinically evident tophi [8]. DECT can help differentiate MSU crystal deposition from other calcifications. US and DECT may provide an alternative to joint aspiration or soft-tissue biopsy in the diagnosis of certain cases of suspected gout, when joint aspiration or soft-tissue biopsy are not available. Goals of management include: (1) treatment and prophylaxis of acute attacks and (2) lowering of serum urate levels with the intent of avoiding flares and suppressing progression of joint damage. Advanced imaging such as US, DECT, and MRI are useful in the assessment of disease burden and response to treatment.
